# East African origins for Madagascan chickens as indicated by mitochondrial DNA

**DOI:** 10.1098/rsos.160787

**Published:** 2017-03-22

**Authors:** Michael B. Herrera, Vicki A. Thomson, Jessica J. Wadley, Philip J. Piper, Sri Sulandari, Anik Budhi Dharmayanthi, Spiridoula Kraitsek, Jaime Gongora, Jeremy J. Austin

**Affiliations:** 1 Australian Centre for Ancient DNA, School of Biological Sciences, University of Adelaide, South Australia, Australia; 2 School of Archaeology and Anthropology, Faculty of Arts, The Australian National University, Canberra, Australian Capital Territory, Australia; 3 Genetic Laboratory, Division of Zoology, Research Center for Biology, The Indonesian Institute of Sciences (LIPI), Jl Raya Jakarta-Bogor Km.46, Cibinong 16911, Indonesia; 4 Centre for Advanced Technologies in Animal Genetics and Reproduction, Faculty of Veterinary Science, University of Sydney, Sydney, New South Wales, Australia

**Keywords:** chicken, human migration, dispersal, Madagascar, mitochondrial DNA

## Abstract

The colonization of Madagascar by Austronesian-speaking people during AD 50–500 represents the most westerly point of the greatest diaspora in prehistory. A range of economically important plants and animals may have accompanied the Austronesians. Domestic chickens (*Gallus gallus*) are found in Madagascar, but it is unclear how they arrived there. Did they accompany the initial Austronesian-speaking populations that reached Madagascar via the Indian Ocean or were they late arrivals with Arabian and African sea-farers? To address this question, we investigated the mitochondrial DNA control region diversity of modern chickens sampled from around the Indian Ocean rim (Southeast Asia, South Asia, the Arabian Peninsula, East Africa and Madagascar). In contrast to the linguistic and human genetic evidence indicating dual African and Southeast Asian ancestry of the Malagasy people, we find that chickens in Madagascar only share a common ancestor with East Africa, which together are genetically closer to South Asian chickens than to those in Southeast Asia. This suggests that the earliest expansion of Austronesian-speaking people across the Indian Ocean did not successfully introduce chickens to Madagascar. Our results further demonstrate the complexity of the translocation history of introduced domesticates in Madagascar.

## 1. Introduction

Beginning in the first few centuries AD, Austronesian speakers from Island Southeast Asia (ISEA) established trade links with India and eventually colonized Madagascar during *ca* AD 50–500 [1,2]. This makes Madagascar the most westerly point of the great Austronesian expansion. Linguistic and genetic evidence [3–6] suggests a dual ancestry for the indigenous people of Madagascar, involving both African and Southeast Asian origins. For example, Malagasy, the language spoken in Madagascar, is a member of the Austronesian language family related to the Barito and Dayak languages spoken in southeast Kalimantan, Indonesia [7]. However, recent genetic studies indicate that contemporary Malagasy populations are derived from genetic admixture involving Indonesian and African ancestors (i.e. Bantu) [5,6]. In addition to genetic and linguistic evidence, transfers of material culture are also evident in the Austronesian-inherited traditions connecting Madagascar to Indonesia [8].

The prehistoric exchanges between Madagascar and its maritime neighbours around the Indian Ocean rim have influenced the current day distribution of many domestic plants and animals in the region [9–11]. The most notable Madagascan domesticates that have originated from ISEA are the taro (*Colocasia esculenta*), Asian yam (*Dioscorea alata*) and banana (*Musa sapientum*) [10]. Their absence in the intervening regions of India and the Arabian Peninsula makes a translocation via a central Indian Ocean maritime corridor [10] more likely than a coastal route [12]. However, genetic studies of ship-borne commensals found in Madagascar, such as rats (*Rattus* sp.), the house mouse (*Mus musculus*) and the shrew (*Suncus murinus*), suggests India as the point of origin [13–15]. An investigation of diversity in domesticate/commensal species and their translocation patterns around the Indian Ocean rim suggests a deeply entrenched trade and contact network [11]. For instance, exchanges in the Arabian Sea led to the movement of certain cereal crops, such as sorghum (*Sorghum bicolor*), pearl millet (*Pennisetum galucum*) and finger millet (*Eleusine coracana*), from Africa to the Arabian Peninsula and India [16], while Zebu cattle were translocated in the reverse direction from India to the Arabian Peninsula and Africa [17]. Translocations across the Bengal Sea included the movements of mung bean (*Vigna raidata*) and horsegram (*Macrotyloma uniflorum*) from India to Southeast Asia [18] and in reverse mango (*Mangifera indica*) and citron (*Citrus medica*) from Southeast Asia to India [19]. The protracted connection between India and the Austronesian speakers in Indonesia probably culminated in the development of the Srivijayan Empire in Indonesia and the Malay Peninsula [20]. The Srivijayan religion, culture and even language contains a mixture of influences not only from India but also from Persia [21]. These instances demonstrate that there has been a long history of contact, trade and exchange between geographically distant groups of people around the Indian Ocean rim. However, uncertainty exists as to how chickens became part of this exchange network.

Chickens were domesticated from jungle fowl (*Gallus* spp*.*) in East Asia during the Mid–Late Holocene [22–24], with subsequent translocation by humans to other parts of the world, including Africa and Madagascar. Similar to banana, taro and yam, chickens are deeply integrated into the subsistence culture of Africa, indicating a certain level of antiquity [25], with chickens first appearing in Madagascar around the late AD eighth–mid nineth century [26]. Archaeological evidence suggests that chickens were introduced into Africa via trade links with Southeast Asia [27]. Furthermore, on Madagascar, the Malagasy term for chicken and other domesticated animals, is borrowed from Bantu languages (from the east coast of Africa), not Austronesian [9,28], suggesting that chickens were initially established on the east coast of Africa before being introduced into Madagascar.

Previous studies have characterized partial mtDNA control region sequences in African village chickens, which fell into two major mitochondrial lineages, that suggest two origins: (i) in Southeast Asia and (ii) the Indian subcontinent [29–31]. Chickens from Madagascar have been reported to also have a dual geographical origin, from continental Africa and Indonesia, on the basis of the same two mitochondrial lineages [32]. However, Razafindraibe *et al.* [32] provided no analysis to support their conclusions.

Thus, the geographical origin of Madagascan chickens remains uncertain, with Africa, South Asia and/or ISEA all possible source populations. Here, we assess the genetic relationships and diversity of chicken populations found around the Indian Ocean rim. We aim to address the question of whether indigenous chickens in Madagascar trace their ancestry to Indonesia as reflected in the expansion of the Austronesian culture, or they resulted from a more complex connection with other areas around the Indian Ocean.

## 2. Material and methods

**Figure 1.**
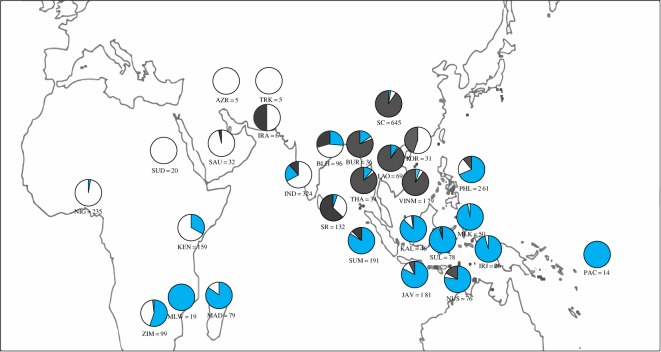
Frequency distribution of chicken mitochondrial DNA haplogroup in the region under study (blue, haplogroup D; white, haplogroup E; grey, other haplogroups) with geographical location and sample size noted. Sample localities are Azerbaijan (AZR), Bangladesh (BLH), Burma (BUR), India (IND), Iran (IRA), Irian Jaya (IRJ), Java (JAV), Kalimantan (KAL), Kenya (KEN), Korea (KOR), Laos (LAO), Madagascar (MAD), Malawi (MLW), Maluku (MLK), Nigeria (NIG), Nusa Tenggara (NUS), Pacific (PAC; Fiji, Solomon and Vanuatu), Philippines (PHL; Luzon, Visayas and Mindanao), Saudi Arabia (SAU), South China (SC), Sri Lanka (SRI), Sudan (SUD), Sulawesi (SUL), Sumatra (SUM), Thailand (THA), Turkmenistan (TRK), Vietnam (VIE) and Zimbabwe (ZIM).

In total, 3128 chicken sequences from Madagascar, Africa, the Arabian Peninsula, West Asia, East Asia, South Asia, ISEA, Mainland Southeast Asia (MSEA) and the Pacific were used for analyses (figure 1; table 1 and the electronic supplementary material, table S1). Mwacharo *et al.* [31] sequenced 512 chickens from East Africa (Kenya: 211, Ethiopia: 43, Sudan: 135, Uganda: 123) but only 159 sequences (all from Kenya) were available on GenBank. We generated new sequences from 898 chickens from ISEA and the Pacific and combined these with 2230 existing sequences (electronic supplementary material, table S1). Seventy-nine previously published sequences were available from Madagascar. Two samples have unknown collection locality; the remaining 77 were collected from two regions in central Madagascar. The sampling approach performed in ISEA and the Pacific was approved by the University of Adelaide Animal Ethics Committee (AEC) as part of the study on reconstructing the human colonization of the Pacific (AEC approval number: S-2011-211). Samples from indigenous village chickens consisted of plucked body feathers. Genomic DNA was extracted from feather samples using the salting-out method [48].

**Table 1.** Details, including collection locality and published source, for 3128 chicken mtDNA control region sequences used in this study. (^#^Mwacharo *et al.* [31] sequenced additional chicken samples from East Africa (Ethiopia: 43, Sudan: 135, Uganda: 123) but did not make them available on GenBank. See the electronic supplementary material, table S1 for detailed attributes of the 3128 sequences.)

region	locality	no. samples	references
Africa^#^			
	Madagascar	79	[32]^b^, [33]^a^
	Malawi	19	[29]
	Zimbabwe	99	[29]
	Kenya	159	[31]
	Nigeria	235	[34]
	Sudan	20	[29]
Arabian Peninsula			
	Saudi Arabia	32	[35]
West Asia			
	Iran	6	[22], [33]^a^
	Azerbaijan	5	[22]
	Turkmenistan	5	[22]
South Asia			
	India	324	[22,23,36]
	Sri Lanka	132	[37]
	Bangladesh	96	[38]
East Asia and Mainland Southeast Asia			
	China	645	[39]^a^, [40]^a^, [22,23,41,42]^a^
	Burma	36	[22,23,43]
	Thailand	34	[44], [33]^a^, [45]
	Laos	69	[33]^a^, [23,46]
	Vietnam	179	[33]^a^, [45], this study
	Korea	31	[47]
Island Southeast Asia			
	Sumatra	191	[44], [33]^a^, [23], this study
	Java	181	[44], this study
	Kalimantan	46	this study
	Nusa Tenggara	76	[33]^a^, [22,46], this study
	Sulawesi	78	this study
	Maluku	50	this study
	Irian Jaya, Papua	26	this study
	Philippines	261	[33]^a^ [46], this study
Pacific			
	Solomon	3	this study
	Vanuatu	9	this study
	Fiji	2	this study

^a^Sequences submitted only to GenBank without publication.

^b^Sequences not submitted to any database, sequences obtained directly from the authors.

A 760 base pair (bp) fragment of the mtDNA control region (CR) was PCR-amplified using primers GallP4F (5′-AACTCCCCTACTAAGTGTACCCCC-3′) and GallP4R (5′-TTGACACTGATGCACTTTGGATCG-3′) from position 43 to 802 in the chicken mtDNA genome (GenBank Accession: X52392 [49]). Each PCR (25 µl final volume) contained 10× Hotmaster buffer, 0.25 mM of each dNTP, 0.2 µM of each primer and 0.1 U of Hotmaster *Taq* DNA polymerase. Thermocycling comprised an initial denaturation and enzyme activation at 94°C for 2 min, followed by 30 cycles of denaturing at 94°C for 20 s, primer annealing at 55°C for 10 s and elongation at 65°C for 60 s, and a final extension at 65°C for 10 m. PCR clean-up and Sanger sequencing were conducted at the Australian Genome Research Facility Ltd. Forward and reverse sequence chromatograms were assembled and manually edited using Geneious 6.5.5 (Biomatters) to obtain a consensus sequence.

DNA sequences, combining newly generated data and existing data, consisted of variable lengths of the mtDNA control region. Sequences were aligned using the MUSCLE algorithm in Geneious v. 6.5.5 and truncated to 349 bp (corresponding to positions 46–394 in the chicken mtDNA genome, GenBank accession no. X52392 [49]), the longest sequence common to all samples. We followed the haplogroup naming system of Miao *et al.* [23], which differs from that of Mwacharo *et al.* [31] and Muchadeyi *et al.* [29]. The number of haplotypes and the number of samples per haplotype were determined by collapsing sequences to unique haplotypes using FaBox 1.40 [50]. Identifying the mitochondrial haplogroup of each unique sequence was done by ordering them into a neighbour-joining tree generated using the Tamura-Nei substitution model and comparing the assignments to those from previously published papers [22,23,51,52].

To assess population genetic differentiation and gene flow among sampling locations, Slatkin's linearized *F*_ST_ was computed using Arlequin v. 3.1 [53] (10 000 permutations). To visualize the relationships between populations, a non-parametric multidimensional scaling plot (MDS) was performed using the Slatkin's linearized *F*_ST_ scores. This was done initially on all chicken mitochondrial haplogroups and then on just haplogroup D, as a substantial proportion of haplotypes found on Madagascar and ISEA belongs to mitochondrial haplogroup D. To further explore geographical structure, an analysis of molecular variance (AMOVA) was also calculated in Arlequin [53] using Indonesia, South Asia, East Africa and Madagascar as groups. These regions were selected for AMOVA because they are the most likely regions involved in the translocation of chickens to Madagascar. Furthermore, the extensive linguistic and human genetic studies mostly involve these regions.

The evolutionary relationships among haplotypes were estimated via a median-joining (MJ) network [54] using Network v. 4.6.1 (fluxus-engineering.com) for haplogroups D and E separately. Pairwise genetic distances between all haplotypes, and again for haplogroup D only, were calculated using Genalex [55] and ordered into principal coordinate analysis (PCoA) plots to further explore the relationships of the lineages and to see how the haplotypes around the Indian Ocean rim are distributed. Intra-population genetic variation and population expansion statistics (i.e. haplotype/nucleotide diversity and Tajima's D/Fu's *F*s, respectively) were also calculated at a regional level using Arlequin v. 3.1 [53].

## 3. Results

### 3.1. Mitochondrial haplogroup distribution patterns

Chickens in Madagascar cluster into two haplogroups: the majority (85%) of samples belong to haplogroup D and the rest belong to haplogroup E (figure 1). Similarly, in East Africa only haplogroup D and E chickens are observed in publically available sequences, with a positive association between haplogroup D frequency and latitude southwards. By contrast, haplogroup D is not observed in chickens from the Arabian Peninsula and western Asia: the haplogroup composition in these regions is dominated by haplogroup E. Within India, haplogroup E chickens are also observed at a high frequency (67%) with haplogroup D (22%) and all other haplogroups (11%) making up the balance. To the east in MSEA, all haplogroups (A to I) are observed, but the frequency of haplogroup D and E are dramatically lower in comparison to other haplogroups (4.8% and 2.4%, respectively). However, chickens from islands further east (in the Pacific Ocean) have a high proportion of haplogroup D (84%).

### 3.2. Population genetic structure

**Figure 2.**
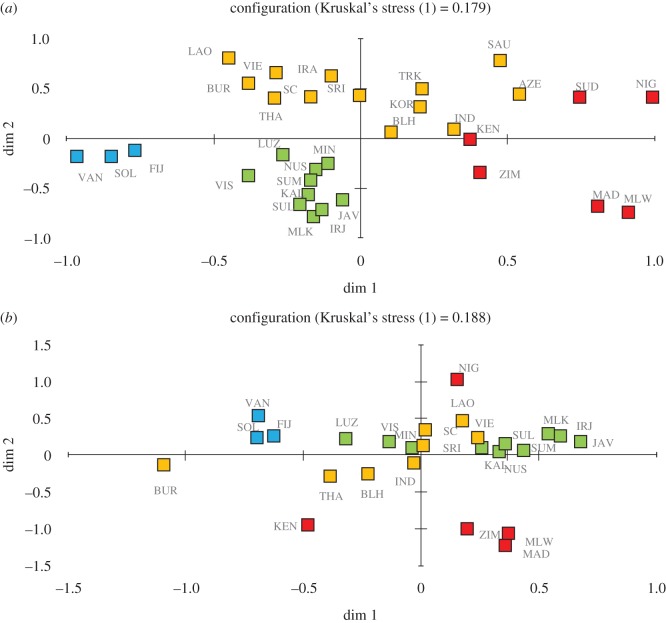
Multidimensional scaling plots (MDS) for pairwise population Slatkin's linearized *F*_ST_ for (*a*) 3128 chickens from Asia (orange), Africa (red), ISEA (green) and the Pacific (blue) using all haplogroups. (*b*) 1081 haplogroup D chickens from the same regions. Azerbaijan, Iran, Korea, Saudi Arabia, Sudan and Turkmenistan are not present in plot B because they do not contain haplogroup D lineages. See figure 1 for locality abbreviations.

The overall genetic structure in the MDS plot using all haplogroups reveals structuring at a broad geographical scale (figure 2*a*). Distinctive clustering of populations from ISEA (shown in green) and from the Pacific (in blue) can be seen. African populations, including Madagascar, sit closer to South and West Asian populations, than ISEA populations. Madagascar falls closest to Malawi, which is the closest African population to Madagascar in the dataset. The other continental African populations show broad geographical clines (northern–southern cline follows high–low ‘Dim 2’ values). When only haplogroup D is used, the Madagascan samples form a distinct cluster with the geographically closest East African populations, Malawi and Zimbabwe (figure 2*b*).

The AMOVA performed on all chickens from Indonesia, South Asia, East Africa and Madagascar show population structure (table 2). However, the among-group variance components (regional groupings) are highest when only haplogroup D chickens are used. For haplogroup D samples, the among-group variance component is always significant when Indonesia is separated from Madagascar. The only non-significant among-group variance component is when South Asia was isolated from Indonesia, East Africa and Madagascar as a group. The variance components among the groups (regional grouping) are generally low when using only haplogroup E.

**Table 2.** Population genetic structure estimated from the analysis of molecular variance (AMOVA) based on mtDNA control region sequences from relevant regions in the Indian Ocean rim: (A) Indonesia, (B) South Asia, (C) East Africa and (D) Madagascar.

				variance components (%)		
group	*n*	no. population	no. groups	among groups	among populations within groups	within populations
haplogroup D and E combined						
no grouping	1347	14	1	…	31.07	68.93
group 1 (A versus B versus C versus D)	1347	14	4	30.38	5.75	63.87
group 2 (A, C and D versus B)	1347	14	2	17.87	19.66	62.46
group 3 (A versus B, C and D)	1347	14	2	23.58	14.77	61.64
group 4 (A, B, versus C, D)	1347	14	2	11.38	24.11	64.51
haplogroup D only						
no grouping	845	14	1	…	48.00	52.00
group 1 (A versus B versus C versus D)	845	14	4	51.98	6.95	41.07
group 2 (A, C, & D versus B)	845	14	2	−*1*.*98*	49.19	52.79
group 3 (A versus B, C and D)	845	14	2	44.25	15.07	40.68
group 4 (A, B, versus C, D)	845	14	2	56.42	9.30	34.28
haplogroup E only						
no grouping	502	10	1	…	9.86	90.14
group 1 (A versus B versus C versus D)	502	10	4	4.88	6.26	88.86
group 2 (A, C and D versus B)	502	10	2	1.80	8.65	89.55
group 3 (A versus B, C and D)	502	10	2	4.78	8.72	86.50
group 4 (A, B, versus C, D)	502	10	2	0.49	9.56	89.96

### 3.3. Population dynamics and genetic variability

The genetic differentiation observed from the *F*_ST_ values (table 3) using both haplogroup D and E samples show that the highest level of divergence is between Indonesia and Madagascar (1.33034), whereas it is the lowest between South Asia and Africa (0.14823). When using only haplogroup D samples, the highest level of divergence is still between Indonesia and Madagascar (0.69607) and the lowest is between Africa and Madagascar (0.13317). All population pairwise comparisons were significant at the 5% level.

**Table 3.** Matrix of Slatkin's linearized *F*_ST_ between chicken samples from Indonesia, South Asia, East Africa and Madagascar based on mitochondrial control region sequences. (*Significant differences at *p *< 0.05.)

population	abbreviation	SA	INDO	AFR	MAD
haplogroup D and E combined					
South Asia	SA	0			
Indonesia	INDO	0.61*	0		
Africa	AFR	0.15*	0.51*	0	
Madagascar	MAD	0.80*	1.33*	0.29*	0
haplogroup D					
South Asia	SA	0			
Indonesia	INDO	0.28*	0		
Africa	AFR	0.38*	0.67*	0	
Madagascar	MAD	0.38*	0.70*	0.13*	0

Both Tajima's D and Fu's *F*s neutrality statistics indicate that chickens from South Asia, Indonesia, East Africa and Madagascar deviate from neutrality when using only haplogroup D (table 4). However, when using both haplogroup D and E, all consistently deviate from neutrality except Madagascar. These results support a model of demographic expansion of haplogroup D for each of the four regions. Furthermore, the highest level of genetic diversity is observed in South Asia and ISEA. On the other hand, Madagascar has the lowest diversity among the four regional groups being compared, with East Africa next lowest, suggesting a genetic bottleneck effect with every new introduction of chickens into an area.

**Table 4.** Genetic diversity measures and historical demographic patterns of chickens from Indonesia, South Asia, East Africa and Madagascar. (*N*(H), size (no. haplotypes); HD, haplotype diversity; ND, nucleotide diversity; *π*, mean no. of pairwise difference; SSD, sum of squared differences. *Statistically significant *p*-values (*p *< 0.05 for Tajima's *D*, *p *< 0.02 for Fu's *F*s).)

	molecular diversity indices				neutrality test	
region	*N*(H)	HD	ND (SD)	*π*	Tajima's *D*	Fu's *F*_S_
haplogroup D and E combined						
Indonesia	583 (115)	0.95	0.0047 (0.0031)	1.63	−2.01*	−27.13*
South Asia	409 (114)	0.91	0.0124 (0.0068)	4.31	−1.97*	−25.10*
Africa	276 (42)	0.91	0.0123 (0.0068)	4.30	−0.03	−17.36*
Madagascar	79 (10)	0.43	0.0048 (0.0032)	1.68	−1.15	−1.88
D haplogroup only						
Indonesia	551 (102)	0.95	0.0038 (0.0026)	1.32	−2.14*	−28.85*
South Asia	100 (40)	0.92	0.0118 (0.0066)	4.13	−1.67*	−25.74*
Africa	127 (19)	0.78	0.0041 (0.0028)	1.41	−1.50*	−11.94*
Madagascar	67 (6)	0.22	0.0008 (0.0009)	0.27	−1.90*	−5.32*

### 3.4. Phylogenetic relationships of Madagascan mtDNA haplotypes in East Africa, South Asia and Indonesia

**Figure 3.**
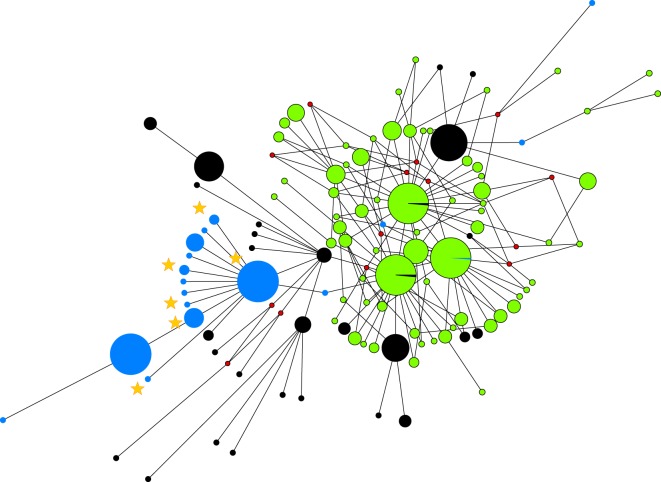
Median-joining network depicting the relationship of D haplotypes of chickens from East Africa and Madagascar (blue), South Asia (black) and Indonesia (green) using all observed D haplotypes regardless of frequency. Stars mark the position of the Madagascan samples. Inferred haplotypes are indicated by small red dots.

**Figure 4.**
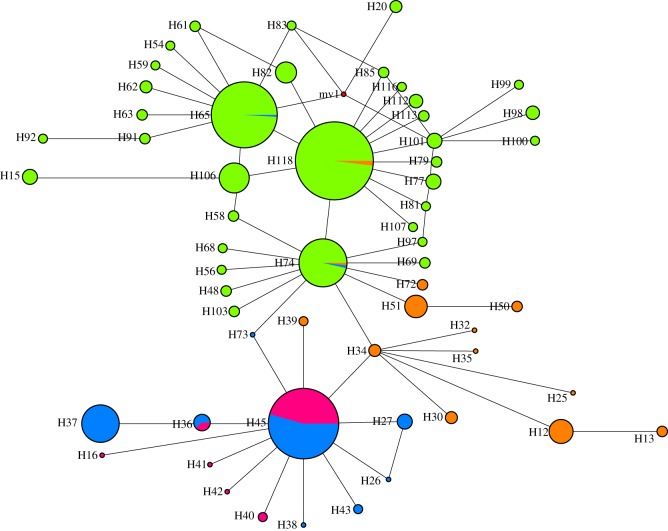
Median-joining (MJ) network of mtDNA-CR D haplotypes observed in Madagascar (purple), Africa (blue), South Asia (brown) and Indonesia (green) excluding most haplotypes represented by one sample. The circle sizes are proportional to the haplotype frequencies and the length of the lines corresponds to the number of mutations connecting haplotypes.

The median-joining phylogenetic network created using only haplogroup D samples show that Madagascar and East Africa share a closely related set of haplotypes and together these two regions have a closer phylogenetic relationship with South Asia than with Indonesia (figure 3). Altogether, there are six Madagascan D haplotypes: H45 and H36 are shared with East Africa and the rest are unique to Madagascar (H16, H40, H41 and H42; figure 4). H45 is the most common D haplotype in East Africa and Madagascar and forms the central node from which the rest of the Madagascan and East African haplotypes radiate. Two predominantly Indonesian haplotypes (H65 and H74) are observed at very low frequencies in continental Africa, but not at all in Madagascar and in fact they are phylogenetically distant to the other Madagascan D haplotypes. The PCoA plot (electronic supplementary material, figure S1) that used the genetic distances between all 112 D haplotypes from Madagascar, East Africa, South Asia and Indonesia also supports the broad separation of regions indicated in the network. Additionally, there are four haplotypes belonging to haplogroup E found on Madagascar. One haplotype is unique to Madagascar, one is shared with East Africa and two are shared with East Africa, South Asia and Indonesia (electronic supplementary material, figure S2).

## 4. Discussion

All our population genetic and phylogeographic analyses of the more than 3000 chicken mtDNA sequences from around the Indian Ocean rim strongly support an African origin for Madagascan chickens. The close relationship between Madagascan and East African chickens is seen in the PCoA plots, the phylogeographic patterns in the networks, as well as in the AMOVA and *F*_ST_ analyses. The progression from high to low diversity and stronger to weaker evidence of demographic expansions from South Asia via East Africa to Madagascar also highlights the effect of repeated genetic bottlenecks as each population was introduced to a new landmass. An African origin for Madagascan chickens is consistent with linguistic evidence—Malagasy words for most domestic animals (dog, goat, cow, sheep, donkey, chicken and guinea fowl) are of Bantu (African) origin [28]. Similarly, Malagasy dogs have an African origin [56] and pig tapeworms in Madagascar trace their origins to South Asia and Africa, not ISEA [57]. This suggests that at least two (dog and chicken) and possibly all three (dog, chicken and pig) animal domesticates successfully transported by Austronesians into the Pacific, were either not transported directly to, or failed to establish, in Madagascar, following the first Austronesian voyages across the Indian Ocean.

The presence of only two haplogroups, D and E, in Madagascar and East Africa suggests that these were the only two lineages translocated to these regions that have survived to the present day, with haplogroup D the dominant lineage in both Madagascar and East Africa. Despite a long and complex history of maritime exchange around the Indian Ocean rim a strong phylogeographic signal remains in haplogroup D chickens (figure 3). The phylogeography indicates a shared common ancestry for Madagascan and East African chickens. The star-like radiation stemming from the most common Madagascar haplotype (H45, which is also the most common east African haplotype; figure 3) suggests that the initial chicken populations arrived in Madagascar via the East African coast. What is not known, however, is whether H45 represents the founding lineage, with *in situ* evolution responsible for the one and two base pair derivations radiating from H45. As the H45 haplotype is currently not observed outside of East Africa/Madagascar and there are no archaeological chicken remains representing the earliest chickens on Madagascar, we cannot tell where H45 originated.

The strong phylogeographic signal within haplogroup D suggests that South Asia, rather than Southeast Asia, is the most likely recent source for East African and Madagascan chickens (figure 3). Therefore, despite the clear linguistic and human genetic associations between Madagascar and Indonesia, the Austronesians do not appear to have successfully translocated chickens directly to Madagascar across the Indian Ocean. However, Austronesians may have transported chickens to Africa indirectly via the Indian subcontinent, rather than via the direct route across the Indian Ocean. Support for this theory can be inferred from the presence of Austronesian speakers in South Asia during the first millennium A.D. [21,58] and the fact that there are no high frequency central nodes in a star-like Indian cluster. Rather, Indian haplotypes belong to two of the high-frequency central nodes in star-like ISEA clusters.

It is interesting that haplogroup D chickens are not observed in the Arabian Peninsula and occur at low frequencies in northeast Africa, suggesting that chickens might have been transported via a direct sea link from India across the Arabian Sea to eastern Africa/Madagascar. Alternatively, Madagascan chickens could also have been transported along a coastal route through the Arabian Peninsula and northeast Africa but with the signal overwritten by subsequent and repeated translocations of haplogroup E chickens.

Haplogroup E is less common in Madagascar compared with haplogroup D. As haplogroup E lacks phylogeographic signature, most probably owing to modern-day translocations (electronic supplementary material, figure S2), it is difficult to derive fine-scale inferences based on haplogroup E other than establishing that it is also most probably South Asian in origin. Furthermore, it is difficult to ascertain whether the arrival of haplogroups D and E was contemporaneous. However, the most parsimonious explanation is that a mixed population of both haplogroup D and E chickens were transported from India to Madagascar via East Africa. Testing this hypothesis is difficult using existing samples. A more deliberate sampling regime, combined with nuclear genetic data and ancient DNA from archaeological samples, would help establish the full history of chicken translocation around the Indian Ocean rim.

## 5. Conclusion

Mitochondrial DNA data suggest that chickens were introduced into Madagascar from South Asia via East Africa. A scenario whereby chickens arrived in Madagascar along with the expansion of the Austronesian-speaking people directly across the Indian Ocean is not supported. However, it remains a possibility that Austronesian traders and mariners integrated South Asian chickens during their coastal voyages *en route* to east Africa and Madagascar. Additional sampling of chickens in Madagascar, increased genomic sequencing of existing samples and accessioning of existing mtDNA data from Mwacharo *et al.* [31] onto publically available databases will help refine our understanding of the ultimate origins of Madagascan chickens.

## Supplementary Material

ESM Figure 1. Principal Coordinate Analysis (PCoA) via covariance matrix of pairwise genetic distances of D haplotypes observed in Africa (blue), South Asia (brown), Indonesia (green), and Madagascar (purple).

## Supplementary Material

ESM Figure 2. Median-joining network depicting the relationship of the E haplotypes observed in East Africa and Madagascar (blue), South Asia (black) and Indonesia (green).

## Supplementary Material

ESM Table 1. Samples used in the study.

## Supplementary Material

ESM Table 2. Genetic diversity measures for each chicken population from Indonesia, South Asia, Continental Africa, and Madagascar.

## Data accessibility

All mtDNA control region sequences generated during this study are available on Genbank under accession numbers KX642436-KX643333 [59].
