# Supplementary material for: East African origins for Madagascan chickens as indicated by mitochondrial DNA
Source: R Soc Open Sci. 2017 Mar 22;4(3):160787. doi: 10.1098/rsos.160787 (PMC5383821; doi:10.1098/rsos.160787)

SI Fig1. Principal Coordinate Analysis (PCoA) via covariance matrix of pairwise genetic distances of D haplotypes observed in Africa (blue), South Asia (brown), Indonesia (green), and Madagascar (purple). Haplotypes found in more than one geographic region are in yellow. Shaded haplotypes in the PCoA corresponds to the haplotypes in the MJ network (inset). Unshaded haplotypes corresponds to haplotypes not shown in the network.

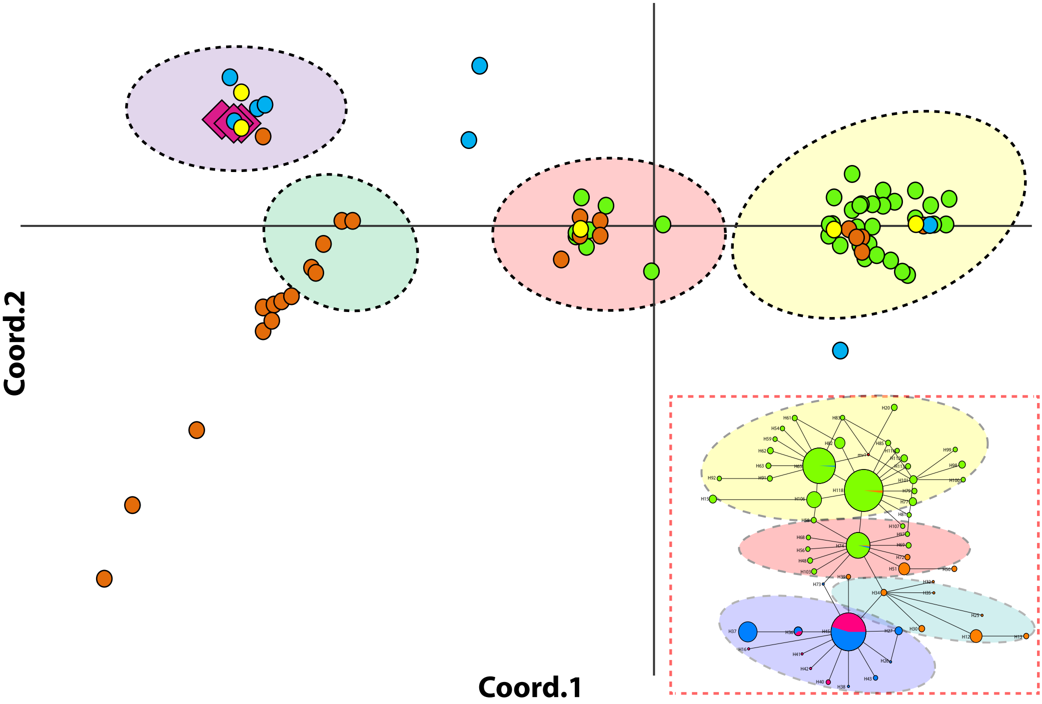

Supplement: ESM Figure 1. Principal Coordinate Analysis (PCoA) via covariance matrix of pairwise genetic distances of D haplotypes observed in Africa (blue), South Asia (brown), Indonesia (green), and Madagascar (purple). [file rsos160787supp1.pdf]
