# Supplementary material for: East African origins for Madagascan chickens as indicated by mitochondrial DNA
Source: R Soc Open Sci. 2017 Mar 22;4(3):160787. doi: 10.1098/rsos.160787 (PMC5383821; doi:10.1098/rsos.160787)

SI Fig2. Median-joining network depicting the relationship of the E haplotypes observed in East Africa and Madagascar (blue), South Asia (black) and Indonesia (green). Stars mark the positions of Madagascan samples. Inferred haplotypes are indicated by small red dots. The inset shows all 80 observed haplotypes.

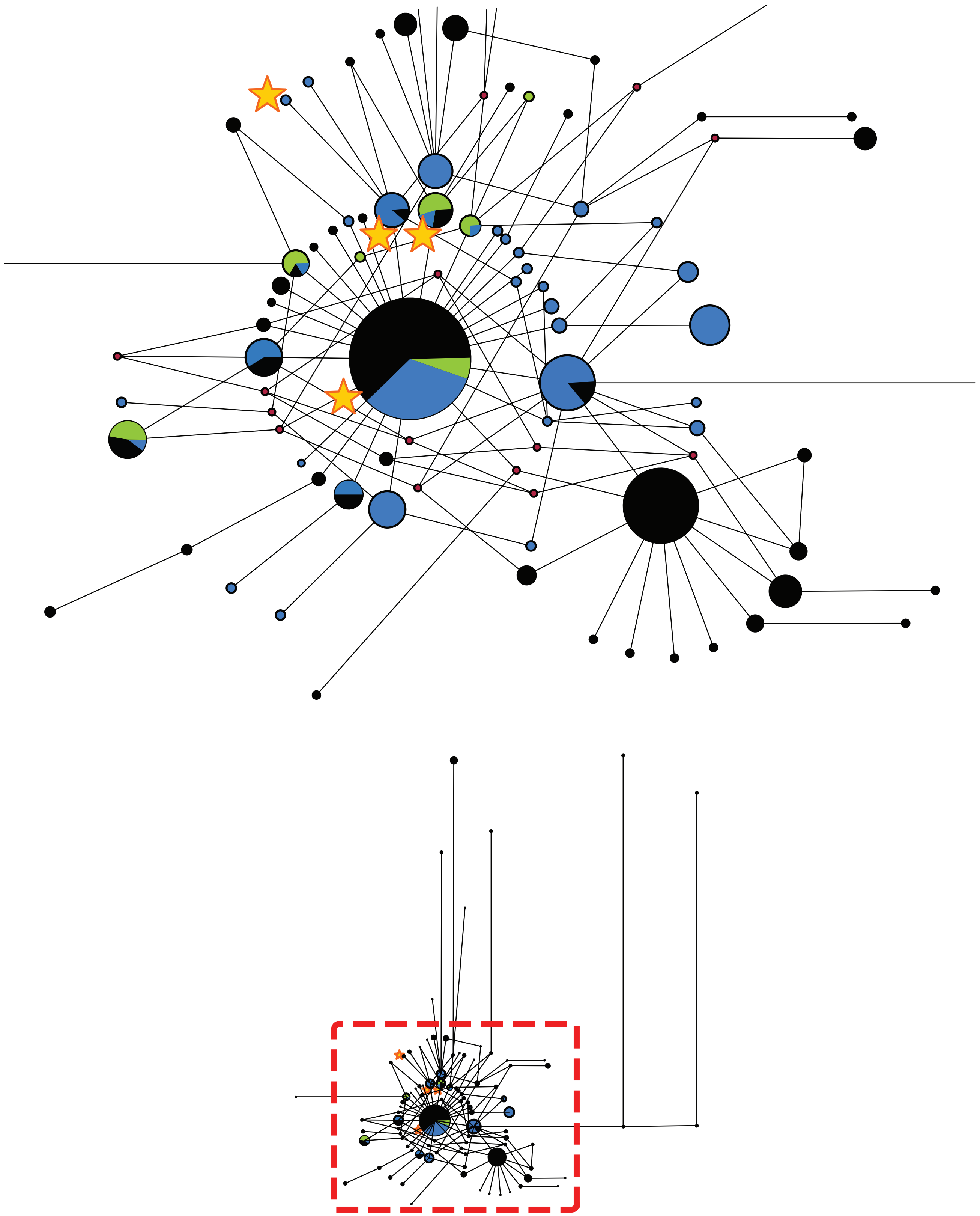

Supplement: ESM Figure 2. Median-joining network depicting the relationship of the E haplotypes observed in East Africa and Madagascar (blue), South Asia (black) and Indonesia (green). [file rsos160787supp2.pdf]
