# Supplementary material for: East African origins for Madagascan chickens as indicated by mitochondrial DNA
Source: R Soc Open Sci. 2017 Mar 22;4(3):160787. doi: 10.1098/rsos.160787 (PMC5383821; doi:10.1098/rsos.160787)
Supplement: ESM Table 2. Genetic diversity measures for each chicken population from Indonesia, South Asia, Continental Africa, and Madagascar. [file rsos160787supp4.docx]

**SI Table 2.** Genetic diversity measures for each chicken population from Indonesia, South Asia, Continental Africa, and Madagascar.

| **Region** | **N(H)** | **HD (SD)** | **ND (SD)** | **π** |
| --- | --- | --- | --- | --- |
| ***All haplogroups*** | | | | |
| **South Asia** | 552 (153) | 0.9494 (0.0063) | 0.0192 (0.0100) | 6.71 |
| India | 324 (90) | 0.8693 (0.0164) | 0.0153 (0.0082) | 5.35 |
| Sri Lanka | 132 (38) | 0.9392 (0.0085) | 0.0208 (0.0108) | 7.24 |
| Bangladesh | 96 (25) | 0.9276 (0.0115) | 0.0202 (0.0106) | 7.06 |
| **Indonesia** | 648 (142) | 0.9602 (0.0030) | 0.0089 (0.0051) | 3.11 |
| Sumatra | 191 (38) | 0.8224 (0.0207) | 0.0115 (0.0064) | 4.01 |
| Java | 181 (35) | 0.7861 (0.0224) | 0.0081 (0.0048) | 2.84 |
| Kalimantan | 46 (13) | 0.8493 (0.0405) | 0.0070 (0.0043) | 2.46 |
| Sulawesi | 78 (23) | 0.8315 (0.0377) | 0.0061 (0.0038) | 2.14 |
| Nusa Tenggara | 76 (21) | 0.8702 (0.0286) | 0.0111 (0.0063) | 3.89 |
| Maluku | 50 (7) | 0.6188 (0.0641) | 0.0032 (0.0023) | 1.10 |
| Irian Jaya | 26 (5) | 0.5662 (0.0862) | 0.0038 (0.0027) | 1.32 |
| **Africa** | 277 (43) | 0.9118 (0.0075) | 0.0123 (0.0068) | 4.32 |
| Kenya | 159 (27) | 0.8505 (0.0171) | 0.0127 (0.0069) | 4.45 |
| Zimbabwe | 99 (13) | 0.7170 (0.0356) | 0.0104 (0.0059) | 3.61 |
| Malawi | 19 (3) | 0.2924 (0.1274) | 0.0009 (0.0011) | 0.30 |
| **Madagascar** | 79 (10) | 0.4340 (0.0680) | 0.0048 (0.0032) | 1.68 |
| ***D haplogroup only*** | | | | |
| **South Asia** | 100 (40) | 0.9166 (0.0186) | 0.0118 (0.0066) | 4.13 |
| India | 71 (31) | 0.8769 (0.0350) | 0.0128 (0.0071) | 4.56 |
| Sri Lanka | 4 (4) | 1.00 (0.1768) | 0.0105 (0.0079) | 3.67 |
| Bangladesh | 25 (5) | 0.6433 (0.0710) | 0.0064 (0.0041) | 2.25 |
| **Indonesia** | 551 (102) | 0.9464 (0.0039) | 0.0038 (0.0026) | 1.32 |
| Sumatra | 154 (25) | 0.7385 (0.0276) | 0.0034 (0.0024) | 1.17 |
| Java | 150 (21) | 0.6915 (0.0259) | 0.0032 (0.0023) | 1.11 |
| Kalimantan | 40 (10) | 0.8077 (0.0507) | 0.0049 (0.0032) | 1.71 |
| Sulawesi | 74 (19) | 0.8127 (0.0407) | 0.0048 (0.0032) | 1.68 |
| Nusa Tenggara | 59 (17) | 0.8101 (0.0452) | 0.0044 (0.0029) | 1.52 |
| Maluku | 49 (6) | 0.6029 (0.0649) | 0.0022 (0.0018) | 0.76 |
| Irian Jaya | 25 (4) | 0.5300 (0.0861) | 0.0021 (0.0018) | 0.74 |
| **Africa** | 127 (19) | 0.7768 (0.0242) | 0.0041 (0.0028) | 1.41 |
| Kenya | 53 (9) | 0.5457 (0.0759) | 0.0038 (0.0027) | 1.32 |
| Zimbabwe | 55 (7) | 0.3003 (0.0802) | 0.0020 (0.0017) | 0.71 |
| Malawi | 19 (3) | 0.2924 (0.1274) | 0.0009 (0.0011) | 0.30 |
| **Madagascar** | 67 (6) | 0.2243 (0.0675) | 0.0008 (0.0009) | 0.27 |

N(H) – size (haplotypes #), HD (SD) – haplotype diversity (standard deviation), ND – nucleotide diversity, π – mean # of pairwise difference , SSD – sum of squared differences, * - statistically significant p-values (p < 0.05 for Tajima’s D, p < 0.02 for Fu’s FS)
